# Remorse‐related suicide attempts among young mothers after COVID‐19 infection

**DOI:** 10.1002/pcn5.116

**Published:** 2023-06-25

**Authors:** Kyohei Otani, Ryohei Yoshikawa, Atsumi Naito, Haruko Fukushima, Kunitaka Matsuishi

**Affiliations:** ^1^ Department of Psychiatry Kobe City Medical Center General Hospital Kobe Hyogo Japan; ^2^ Department of Psychiatry Kakogawa Central City Hospital Kakogawa City Hyogo Japan

**Keywords:** suicide, young women, mother, COVID‐19, remorse, Japan

## Abstract

**Background:**

In Japan, there is a tendency to view COVID‐19 infection as one's own responsibility, which may result in more feelings of guilt than in other countries. During the COVID‐19 pandemic, the curfew imposed by COVID‐19 restricted social behavior and increased anxiety and loneliness, which may have increased the risk of suicide among young women, especially mothers who were highly stressed regarding COVID‐19 infection in their children.

**Case Presentation:**

This is a case report of two Japanese mothers who developed feelings of guilt following infection with COVID‐19, leading to suicide attempts. They feared stigma or denial due to the infection, which they were unable to explain to others, leading to a heightened sense of self‐blame and suicide attempts. In addition, Japanese women have a heavy burden of housework, despite their dual roles at home and at work; the pandemic's behavioral restrictions led to increased time at home and stress. These women were also more affected by the economic crisis in the early stages of the pandemic than men. Relatedly, neuropsychiatric symptoms that persisted after recovering from COVID‐19, such as depression, anxiety, fatigue, and pain, namely postacute COVID‐19 syndrome or long COVID, may have precipitated the suicidal ideation in these cases. Moreover, the complication of bipolar disorder by COVID‐19 could have led to suicide attempts caused by infection‐related neuropsychiatric symptoms and the exacerbation of the bipolar disorder by restrictions imposed during the pandemic.

**Conclusion:**

Suicide prevention measures need to be taken more seriously among mothers during or after the COVID‐19 pandemic.

## BACKGROUND

**Figure 1:**
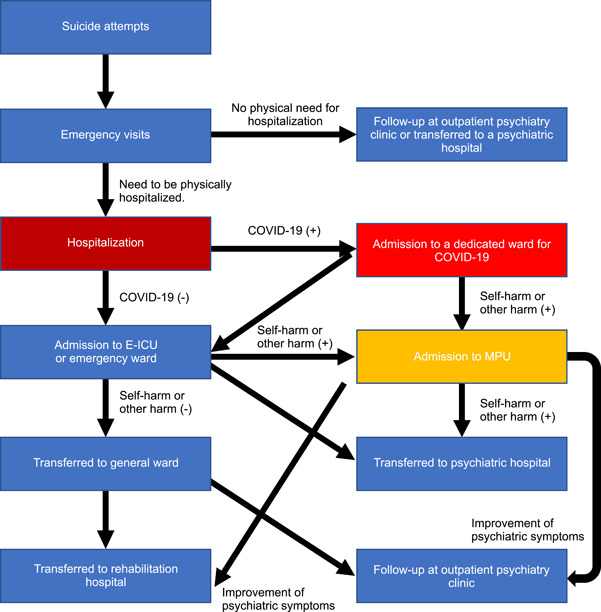
The flow of hospitalized patients with suicide attempts at Kobe City Medical Center General Hospital. E‐ICU, emergency intensive care unit; MPU, Medical Psychiatric Unit.

Rates of suicide are reportedly on the rise after the COVID‐19 pandemic, especially among young women in Japan.^1^ The number of suicides among women in Japan in fiscal year 2020 was 31% higher than predicted; the number of suicides reportedly increased significantly, by 72%, among those aged in their 20s.^2^ Reasons for suicide among women are speculated to include unemployment and caregiving roles, indicating that the pandemic may have placed a strain on several aspects of their lives. School closures, telecommuting, increased caregiver roles, and limited access to healthcare services may be associated with women spending more time with their families.^3^ Women are more susceptible to depression and anxiety disorders, and the COVID‐19 pandemic has exacerbated existing inequalities, such as the increasing and intensifying violence against women.^4^ Japanese women are not only expected to work but also to play the role of the mother at home, which may have become a burden for women in the past. In the first 2 years of the pandemic, 101 patients were admitted to Kobe City Medical Center General Hospital for suicide attempts. Of these, 23 patients (22.8%) entered the eight‐bed medical psychiatric unit (MPU), including two post‐COVID‐infected patients (Table 1). Figure 1 shows the flow of hospitalized patients with suicide attempts at Kobe City Medical Center General Hospital.

**Table 1:** Characteristics comparison of patients admitted to Kobe City Medical Central General Hospital's Medical Psychiatric Unit (MPU) or others for suicide attempts and examined by psychiatrists during the COVID‐19 pandemic (April 1, 2020 to March 31, 2022).

	**Other unit (** ***N*** **= 78)**		**MPU (** ***N*** **= 23)**		***P*‐value**
Age, mean [SD] (years)^a^	46.0 [24.0]		44.0 [20.0]		0.685
Age, range (years)	15–90		14–76		
Female sex, *n* (%)^b^	44	56.4%	14	60.9%	0.812
Occupation					
Unemployed, *n* (%)^c^	45	57.7%	10	43.5%	0.244
Employed, *n* (%)^c^	33	31.0%	13	56.5%	0.244
Student, *n* (%)^c^	13	16.7%	4	17.4%	1.00
Skilled/technical, *n* (%)^c^	9	11.5%	2	13.0%	1.00
Office worker, *n* (%)^c^	6	12.0%	3	8.9%	0.422
Heathcare worker, *n* (%)^c^	3	7.7%	2	8.7%	0.319
Others, *n* (%)^c^	2	2.6%	2	8.7%	0.222
Cohabitants, *n* (%)^c^	39	50.0%	14	60.9%	0.477
Psychiatric history					
Depression, *n* (%)^c^	12	15.4%	6	26.1%	0.351
Bipolar disorder, *n* (%)^c^	7	9.0%	4	17.4%	0.266
Schizophrenia, *n* (%)^c^	6	8.0%	4	17.4	0.229
Insomnia, *n* (%)^c^	5	5.1%	0	0.0%	0.586
Anxiety disorder, *n* (%)^c^	4	6.0%	0	0.0%	0.571
Personality disorder, *n* (%)^c^	2	2.6%	1	4.3%	0.543
Dissociative disorder, *n* (%)^c^	2	2.6%	1	4.3%	0.543
Mental retardation, *n* (%)^c^	0	0.0%	3	13.0%	0.011^a^
Others, *n* (%)^c^	9	11.5%	3	13.0%	1.00
None, *n* (%)^c^	29	24.0%	1	4.3%	0.002^a^
Psychotropic medication history					
Antipsychotic drug use, *n* (%)^c^	32	41.0%	14	60.9%	0.103
Antidepressant use, *n* (%)^c^	16	20.5%	5	20.8%	1.00
Anxiolytic and sleep medication (benzodiazepines), *n* (%)^c^ use	48	61.5	13	21.7%	0.809
History of suicide attempts, *n* (%)^c^	40	57%	7	30.4%	0.098
Physical comorbidities, *n* (%)^c^	33	51.3%	5	21.7%	0.090
Charlson comorbidity index, points [SD]^a^	0.42 [1.01]		0.30 [1.26]		0.681
COVID‐19 infection, *n* (%)^c^	4	5.1%	2	8.7%	0.617
Psychiatric diagnosis at admission					
Adjustment disorder, *n* (%)^c^	19	24.4%	0	0.0%	0.006^a^
Schizophrenia, *n* (%)^c^	10	12.8%	3	13.0%	1.00
Depression, *n* (%)^c^	9	11.5%	5	21.7%	0.300
Alcoholism, *n* (%)^c^	9	11.5%	1	4.3%	0.448
Mental retardation, *n* (%)^c^	8	10.3%	0	0.0%	0.193
Personality disorder, *n* (%)^c^	8	10.3%	0	0.0%	0.193
Bipolar disorder, *n* (%)^c^	7	9.0%	4	17.4%	0.266
Dissociative disorder, *n* (%)^c^	6	7.7%	0	0.0%	0.332
Others, *n* (%)^c^	10	12.8%	2	8.7%	0.729
Diagnosis on admission					
Acute drug intoxication, *n* (%)^c^	67	85.9%	4	17.4%	<0.001^a^
(Multiple) bone fracture, *n* (%)^c^	4	5.1%	11	47.8%	<0.001^a^
Cut wound (chest, abdomen, neck), *n* (%)^c^	6	7.7%	6	26.1%	0.027^a^
Drowning, *n* (%)^c^	0	0.0%	1	4.3%	0.228
Others, *n* (%)^c^	0	0.0%	1	4.3%	0.228
None, *n* (%)^c^	1	1.3%	0	0.0%	1.00
Outcome					
Home, *n* (%)^c^	58	74.4%	8	34.8%	0.001^a^
Transferred to psychiatric hospital, *n* (%)^c^	12	15.4%	7	30.4%	0.131
Transferred to rehabilitation hospital, *n* (%)^c^	8	10.2%	8	34.8%	0.009^a^

*Note*: All information was obtained from medical record databases.

The Charlson comorbidity index was calculated using records from the Japanese Diagnosis Procedure Combination (DPC) data. COVID‐19 negative: 95 patients without COVID‐19 were inpatients for suicide attempts and were attended to by psychiatrists during the same period.

Abbreviation: SD, standard deviation.

^a^ Analyzed by *t*‐test.

^b^ Analyzed by *χ* ^2^ test. Statistically significant difference (*P* < 0.05).

^c^ Analyzed using Fisher's exact probability test.

This brief report describes the cases of two mothers aged in their 30s who attempted suicide after contracting COVID‐19. One patient reported first‐episode depression, while the other had bipolar disorder with a depressive phase, which led to her suicidal behavior. They feared stigma or treatment denial due to the infection or because they were unable to explain the cause of the infection to others; this led to a heightened sense of self‐blame and, eventually, their suicide attempts.

## CASE PRESENTATION

### Case 1

Ms. K.I., a 38‐year‐old female with no psychiatric history, living with her husband and three children, was a childcare worker who feared COVID‐19 infection at work. In addition, due to the closure of her workplace and her children's school classes, she spent more time at home with her family and more time doing household chores, which caused her to feel stressed. She presented to the emergency department with multiple bone fractures. After the COVID‐19 infection, despite mild symptoms, including mild fever, cough, malaise, and odor and taste disturbances, she started feeling increased self‐doubt and hopelessness and later recalled taking her children to the pediatrician and visiting the library. She confided that she lied about her oldest son being ill. She was afraid that her lies might have resulted in her infecting people with COVID‐19 with whom she came into close contact. Out of remorse, she jumped from the sixth floor, resulting in a suicide attempt. She tested negative for COVID‐19 but complained of fatigue, agitation, loss of taste, smell, and appetite, and depressive mood. She was administered 10 mg olanzapine and 5 mg escitalopram, but self‐harm attempts to fall out of bed, agitation, and screaming were continuously observed. She was voluntarily admitted to the MPU on hospital day (HD) 12. With the quiet environment and the active involvement of the medical staff, her insomnia, anxiety, and agitation improved, and she was transferred to the general ward and then discharged home. Her taste and smell disorders recovered, and she returned to work.

### Case 2

Ms. K.Y., a 33‐year‐old female, a natural worrier, and living with her son and parents, was taken to the emergency room with multiple fractures. After graduating from junior college, she moved from one job to another. She had been attending various mental health clinics and experienced a strong contrast between mania and depression. She was married and gave birth to a child but was currently divorced. She was unable to get a regular job and lived with her elderly parents and son, which strongly manifested her fear of infection as she did not want to infect her parents. Furthermore, she suffered anxiety about her finances as she lost her job at the start of the pandemic. When she and her son tested positive for COVID‐19, she worked without telling anyone the truth, fearing that “the police will catch me.” A depressed mood, self‐blame, and feelings of guilt were observed. Even with only mild COVID‐19 symptoms of fever and fatigue, she attempted to jump to her death. On admission, she was administered 40 mg lurasidone and, on HD 10, was admitted to the MPU involuntarily. She initially calmed down, but her agitation grew gradually as her self‐doubt intensified. She attempted to strangle herself using a call button cord. Subsequently, she was transferred to a psychiatric hospital to improve her symptoms of suicide ideation, agitation, and depression.

## DISCUSSION

We present two cases of young mothers who developed feelings of guilt following infection with COVID‐19, leading to attempted suicide. Case 1 was a first‐episode depression and showed taste and odor disturbances, which were suspected to be effects of the COVID‐19 infection. In Case 2, the bipolar disorder was a preexisting condition, which may have been exacerbated by the infection and by the restrictions imposed during the pandemic. Indeed, among severe mental illnesses, bipolar disorder has particularly been associated with an increased risk of suicide.

**Figure 2:**
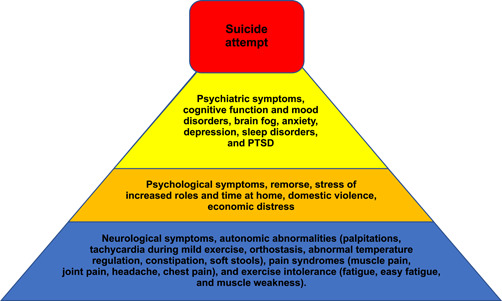
Model diagram of factors leading to suicide attempts in a pandemic. PTSD, post–traumatic stress disorder.

Neuropsychiatric symptoms, persistent fatigue, and cognitive impairment, including delirium,^5^ can occur following the resolution of acute COVID‐19.^6^ Cognitive function and mood disorders, such as brain fog, anxiety, depression, sleep disorders, and posttraumatic stress disorder; autonomic abnormalities, such as palpitations, tachycardia during mild exercise and orthostasis, abnormal temperature regulation, constipation, and soft stools; pain syndromes, such as muscle pain, joint pain, headache, and chest pain; and exercise intolerance, such as fatigue, easy fatigue, and muscle weakness^7^ can persist after testing negative for COVID‐19 infection, which is known as postacute COVID‐19 syndrome^8^ or long COVID.^9^ Such disorders may have precipitated the acute psychiatric symptoms in these cases (Figure 2).^10^

Cytokine storms associated with hyperinflammation to the infection can lead to persistent neuroinflammation, which suggests a link between long COVID and psychotic symptoms and suicide‐related behaviors.^11^ Psychological symptoms, such as remorse; psychiatric symptoms, such as depression and anxiety; and neurological symptoms, including fatigue and pain, may have combined to produce suicidal ideation.^12^ Regarding Case 2, the preexisting bipolar disorder may have been exacerbated by the COVID‐19 infection. Patients with severe psychiatric disorders are known to be susceptible to COVID‐19 infection,^13^ and mood disorders are associated with a high risk of hospitalization and death.^14^ COVID‐19 exacerbates psychiatric symptoms even 2 years after infection.^15^ It has also been suggested that bipolar disorders are associated with increased suicide rates during a pandemic.^16^

Thus, family physicians should examine and screen those with preexisting psychiatric disorders more carefully. Prevention of COVID‐19 infection, vaccination, infection control measures, and mental healthcare are necessary for infected persons. Suicide prevention measures need to be taken more seriously among mothers, especially during and after the COVID‐19 pandemic.

Additionally, in Japan, there is a tendency to view COVID‐19 infection as one's own responsibility, which may lead to feelings of guilt, compared to other countries. During the COVID‐19 pandemic, lockdown restrictions inhibited social behavior, thereby increasing anxiety and loneliness, which may increase the risk of suicide, especially among young people.^17^ In Europe and the United States, when an epidemic breaks out, the government imposes a ban on gatherings; however, in Japan, being a country of self‐regulation, there is no need to do so. Japanese people are attentive and considerate of their surroundings and traditionally have a group consciousness and group cohesion that distinguishes between natives and foreigners. This has contributed to discrimination against people who have, or are suspected of having, COVID‐19.^18^ Additionally, mothers have been reported to be highly stressed about their children becoming infected,^19^ and we can imagine the greater stress that would be experienced by a mother infecting her child. It is quite understandable that a mother with a child would attempt suicide in the wake of COVID‐19 infection.^20^

Another important point is the economic problems experienced by women. During the pandemic, jobs such as hospitality and food service were severely reduced. Women were more often engaged in these occupations, which is why the number of unemployed women increased compared to men on entering the pandemic in Japan. Women therefore faced greater economic hardship than men. Japanese women's time required for childcare increased, while women's time for housework did not change. The current situation of Japanese women is that they have to work, although the time they require for housework and childcare is twice that of men.^21^ During a pandemic, it is necessary to consider shifting to growing industries, such as medical care, welfare, nursing care, and information and telecommunications, and to develop human resources and improve the working environment. It is also important to promote a paternity leave system for men and decrease the burden of housework and childcare for women.

During the consultation, the psychiatrist should try to listen to the female patient's work concerns and financial concerns, and provide advice on the burden of spending more time with her children and husband. It is also important to check for domestic violence, if necessary.^22^ In addition, it is important to provide continuous support for those that attempt suicide; at our hospital, liaison nurses provide continuous support intervention during hospitalization for those who have been admitted following a suicide attempt. This has been recognized as effective based on a previous study (ACTION‐J)^23^ and is reimbursed as a psychiatric continuous support fee for emergency patients. In the outpatient clinic, psychologists follow up with patients who have attempted suicide as a joint project with the Kobe City Mental Health Welfare Center, which is free of charge as a municipal project. In the unfortunate event of suicide, Kobe City introduces volunteer suicide survivors' associations to provide mental healthcare support for suicide survivors.^24^

In contrast, it has been reported that the suicide rate has decreased among the older population^25^ and in the areas affected by the Great East Japan Earthquake.^26^ However, our comparative study of pre‐ and during‐COVID‐19 suicide attempts in the Hanshin area found no significant increase.^27^ People in this region experienced disasters such as the Great Hanshin‐Awaji Earthquake and the H1N1 influenza, which may have contributed to a greater experience in disaster preparation, infection control, and mental healthcare. This suggests that suicide prevention measures for high‐risk groups may have been effective during the COVID‐19 pandemic.

## CONCLUSION

Young women, especially mothers, who are infected with COVID‐19 in Japan require additional mental healthcare and follow up to prevent suicide.

## AUTHOR CONTRIBUTIONS

Kyohei Otani, Ryohei Yoshikawa, Atsumi Naito, Haruko Fukushima, and Kunitaka Matsuishi were involved in the study design. Ryohei Yoshikawa, Atsumi Naito, and Kunitaka Matsuishi contributed to the data analysis. Haruko Fukushima and Kyohei Otani contributed to the acquisition of data. Kyohei Otani drafted the initial manuscript, which was then revised by Haruko Fukushima. All authors approved the final manuscript.

## CONFLICT OF INTEREST STATEMENT

The authors declare no conflict of interest.

## ETHICS APPROVAL STATEMENT

This study was conducted in accordance with the principles of the Declaration of Helsinki and was approved by the Institutional Review Board of the Kobe City Medical Center General Hospital.

## PATIENT CONSENT STATEMENT

Written consent for case reports was obtained from the patients. The opt‐out method was used to obtain informed consent.

## CLINICAL TRIAL REGISTRATION

N/A.

## Data Availability

The data that support the findings of this study are available from the corresponding author upon reasonable request.
